# Conversion of Lithium Chloride into Lithium Hydroxide by Solvent Extraction

**DOI:** 10.1007/s40831-022-00629-2

**Published:** 2022-12-05

**Authors:** Viet Tu Nguyen, Clio Deferm, Ward Caytan, Sofía Riaño, Peter Tom Jones, Koen Binnemans

**Affiliations:** 1 Department of Chemistry, KU Leuven, Celestijnenlaan 200F, Box 2404, 3001 Leuven, Belgium; 2 Department of Materials Engineering, KU Leuven, Kasteelpark Arenberg 44, Box 2450, 3001 Leuven, Belgium

**Keywords:** Aliquat 336, Antisolvent precipitation, Hydrometallurgy, Ion exchange, Lithium, Solvent extraction

## Abstract

A hydrometallurgical process is described for conversion of an aqueous solution of lithium chloride into an aqueous solution of lithium hydroxide via a chloride/hydroxide anion exchange reaction by solvent extraction. The organic phase comprises a quaternary ammonium chloride and a hydrophobic phenol in a diluent. The best results were observed for a mixture of the quaternary ammonium chloride Aliquat 336 and 2,6-di-*tert*-butylphenol (1:1 molar ratio) in the aliphatic diluent Shellsol D70. The solvent extraction process involves two steps. In the first step, the organic phase is contacted with an aqueous sodium hydroxide solution. The phenol is deprotonated, and a chloride ion is simultaneously transferred to the aqueous phase, leading to in situ formation of a quaternary ammonium phenolate in the organic phase. The organic phase, comprising the quaternary ammonium phenolate, is contacted in the second step with an aqueous lithium chloride solution. This contact converts the phenolate into the corresponding phenol by protonation with water extracted to the organic phase, followed by a transfer of hydroxide ions to the aqueous phase and chloride ions to the organic phase. As a result, the aqueous lithium chloride solution is transformed into a lithium hydroxide solution. The process has been demonstrated in continuous counter-current mode in mixer–settlers. Solid battery-grade lithium hydroxide monohydrate was obtained from the aqueous solution by crystallization or by antisolvent precipitation with isopropanol. The process consumes no chemicals other than sodium hydroxide. No waste is generated, with the exception of an aqueous sodium chloride solution.

**Graphical Abstract:**

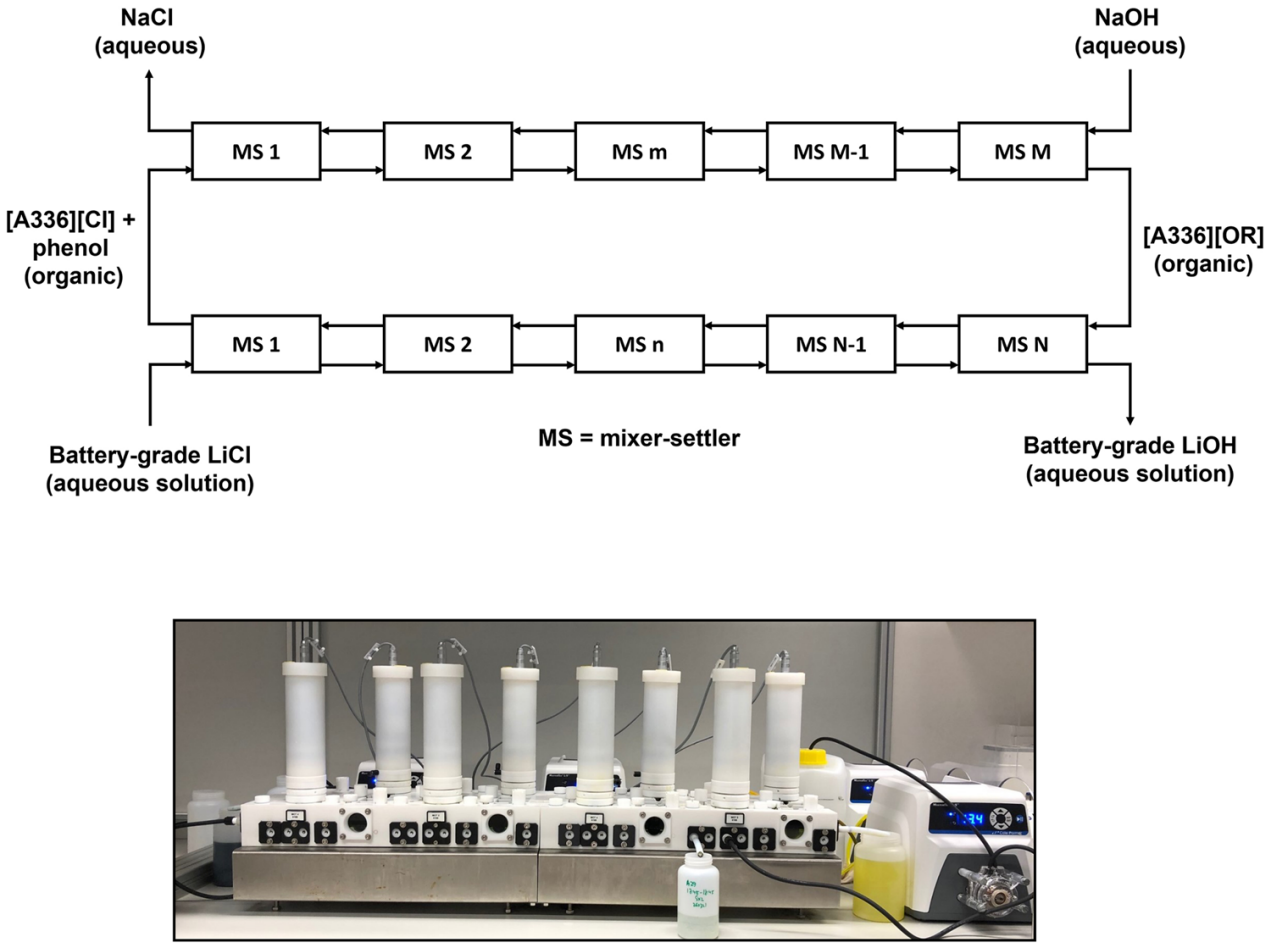

**Supplementary Information:**

The online version contains supplementary material available at 10.1007/s40831-022-00629-2.

## Introduction

Lithium is an indispensable raw material for lithium-ion batteries (LIBs) [1, 2]. At present, lithium carbonate (Li_2_CO_3_) is still the predominant lithium compound for LIB production. Nevertheless, the importance of lithium hydroxide (LiOH) is rapidly increasing, as NMC cathode materials (NMC = nickel –manganese–cobalt) become richer in nickel. Indeed, if the metals are used in a ratio of 6 parts of nickel to 2 parts of cobalt and 2 parts of manganese (6–2–2), or in a 8–1–1 composition, rather than the 1–1–1 or 5–3–2 compositions as in the past, the synthesis requires LiOH rather than Li_2_CO_3_ [3]. The higher temperature required to synthesize nickel-rich cathode materials with Li_2_CO_3_ damages the crystal structure of the cathode and changes the oxidation state of nickel [4], whereas LiOH allows fast and complete synthesis at lower temperatures [5]. Lithium hydroxide is available on the market in the form of its monohydrate LiOH·H_2_O. Battery-grade LiOH·H_2_O has a purity of at least 99.5%. This change in demand of lithium salts is shifting lithium-mining projects towards developing LiOH production rather than Li_2_CO_3_. Industrial production of LiOH is conventionally conducted in a number of ways including (1) reaction of Li_2_CO_3_ with Ca(OH)_2_ and rejection of CaCO_3_ [6, 7], (2) reaction of Li_2_SO_4_ with NaOH and rejection of Na_2_SO_4_ [8], (3) reaction of Li_2_SO_4_ with Ba(OH)_2_ and rejection of BaSO_4_ [9], and (4) direct alkali processing of spodumene [10–12].

Although lithium chloride (LiCl) cannot be directly used in LIBs, it is a valuable intermediate since it can be converted into LiOH. Different methods are known to extract LiCl from different lithium-containing raw materials. These sources include hard-rock lithium ores (pegmatites comprising spodumene, petalite, lepidolite, amblygonite, triphylite, and other lithium minerals), salt lake brines, geothermal brines, and lithium-containing metallurgical slags from pyrometallurgical recycling processes of end-of-life lithium-ion batteries. The technical-grade solid LiCl obtained from these different resources contains different types and concentrations of impurities, depending on the type of material and the source. The main impurities are the alkali metal chlorides NaCl and KCl, and the alkaline earth-metal chlorides MgCl_2_ and CaCl_2_. LiCl can be prepared from spodumene by chlorination roasting with CaCl_2_ [13–15]. Direct chlorination of spodumene by chlorine gas gives results similar to those of CaCl_2_ roasting [16]. Lithium hard-rock ores other than spodumene can be treated via processes similar to those used for spodumene. LiCl can also be prepared from metallurgical slags—originating from pyrometallurgical operations for recycling of LIBs—by processes similarly to those used for the recovery of LiCl from spodumene [17]. Here, LiCl can be fumed from the molten slag by the addition of alkali or earth alkali chlorides [18]. Another LiCl source can be derived through *direct lithium extraction* (DLE) from brines and other lithium-containing solutions [19]. DLE is based on the use of lithium-selective solid adsorbents [20–23]. Subsequently, the adsorbed lithium can be released from the adsorbents by elution with an HCl solution, yielding a highly pure aqueous LiCl solution.

Recently, we described a method for purification of technical LiCl to battery-grade LiCl by a solvometallurgical process that is based on differences in solubility of LiCl compared to other alkali chlorides and alkaline earth chlorides in organic solvents [24]. A process for direct transformation of the obtained battery-grade LiCl into LiOH·H_2_O, bypassing the Li_2_CO_3_ intermediate, would be advantageous from an economical and environmental point of view. Such a process would reduce the number of processing steps, while consuming less chemicals and generating less waste. Conversion of LiCl into LiOH can be achieved by *membrane electrolysis* via a process that is similar to the production of NaOH from NaCl [25, 26]. Typically, a fluorinated cation-exchange membrane with sulfonic acid groups is being used (e.g., Nafion membrane). The main drawbacks of the membrane electrolysis process are the high price of membranes, the loss in energy efficiency due to the internal resistance of the ion-exchange membrane and the fact that the membranes are susceptible to fouling and scaling. When LiCl is used as electrolyte, toxic Cl_2_ gas is released at the anode during electrolysis. Moreover, the Cl_2_ gas can also attack the membrane. *Membrane electrodialysis* is a technique that is similar to membrane electrolysis [27, 28]. The same drawbacks of membrane electrolysis are applied to electrodialysis.

Direct chloride-to-hydroxide conversion by solvent extraction or a similar technique would have several advantages with respect to membrane electrolysis or ion exchange for the direct conversion of LiCl to LiOH. However, chloride/hydroxide anion exchange via solvent extraction is challenging because of difficulties in preparing a solvent phase with a basic extractant (liquid anion exchanger) in the hydroxide form and the limited stability of such compounds due to the Hofmann elimination reaction [29–31]. Sasson and co-workers described a process for conversion of metal chlorides and quaternary ammonium chlorides into the corresponding hydroxide salts by a liquid membrane process comprising a quaternary ammonium chloride and an aliphatic alcohol in hexane [32, 33]. The mechanism is described as a transfer of hydroxide ions across the liquid membrane. The authors state that the very weak acidity of the alcohol is essential (p*K*_a_ > 16), and that extraction efficiency depends on the type of alcohol, in the order: diols > primary alcohols > secondary alcohols. The authors mention that the driving force is the concentration gradient, so that they use a very high NaOH concentration in the aqueous phase in the first exchange step (at least 2.5 mol kg^−1^, but preferably 10 to 12.5 mol kg^−1^). This process was demonstrated for conversion of KCl to KOH, but it was found to be inefficient for conversion of LiCl into LiOH: after 7 contact cycles, only 65% of the chloride ions had been replaced by hydroxide ions in a LiCl solution (21% w/w, 5 mol kg^−1^), by using a 40% w/w (10 mol kg^−1^) NaOH solution.

In this paper, we describe a novel solvent extraction process for conversion of LiCl into battery-grade LiOH·H_2_O, based on solvent extraction (SX) and antisolvent precipitation. No chemicals other than NaOH are consumed and the only waste comprises an aqueous NaCl solution. The process is demonstrated in continuous counter-current mode in mixer–settlers.

## Experimental

### Chemicals

Lithium chloride (> 99%), sulfuric acid (> 95%), 1-octanol (99%), 1-decanol (98 + %), 2-ethyl-1,3-hexanediol (99%), 4-*tert*-butylphenol (97%), 2,6-dimethylphenol (99%), and methanol for HPLC (99.8%) were purchased from Thermo Fisher Scientific (Geel, Belgium). Aliquat® 336 (90.6% quaternary ammonium chloride content, [A336][Cl]), nonylphenol (technical grade, mixture of ring and chain isomers), 2,6-di-*tert*-butylphenol (99%), 2,4,6-tri-*tert*-butylphenol (96%), *p*-cresol (99%), and the ICP standards of 1000 ppm Li, and Na in 2 wt% HNO_3_ were purchased from Sigma–Aldrich (Overijse, Belgium). 2-Methyl-2,4-pentanediol (99%), 2,5-dimethyl-2,5-hexanediol (97%), and 2,2,4-trimethyl-1,3-pentanediol (97%) were from Merck (Overijse, Belgium). 1-Pentanol (> 99%) was obtained from Riedel-de Haën, 1-hexanol (> 98%) was from Fluka, and 2-ethyl-1-hexanol (99%) was from Alfa Aesar (Merelbeke, Belgium). Isopropanol (2-propanol), silver nitrate (volumetric, 0.05 M), nitric acid (volumetric, 0.1 M), and sodium hydroxide (40 vol% solution) were purchased from Chem Lab (Zedelgem, Belgium). Trihexyl(tetradecyl)phosphonium chloride (Cyphos® IL 101) was secured from Cytec Industries Inc. (Niagara Falls, Ontario, Canada). Shellsol D70 (aliphatic diluent), Shellsol A150 (aromatic diluent), and Shell GTL Solvent GS190 (aliphatic diluent) were obtained from Shell Global Solutions (Amsterdam, The Netherlands). All chemicals were used as received, without any further purification.

### Analytical Procedures

The composition and the purity of Aliquat 336, [A336][Cl], were determined by an Agilent liquid chromatography–mass spectrometry system (LC–MS), comprising an Agilent 1100 HPLC system in combination with an Agilent 6110 SQ mass spectrometry system. Aliquat 336 was diluted in methanol to a concentration of 0.1 g/L and analyzed in duplicate. The Aliquat 336 had a quaternary ammonium content of 90.6%. It was composed out of 30.7% of trioctylmethylammonium chloride, 39.7% of dioctyldecylmethymammonium chloride, 23.5% of octyldidecylmethylammonium chloride, and 6.0% of tridecylammonium chloride. Based on the established composition of Aliquat 336, an average molecular weight of 432 g mol^−1^ was calculated.

The chloride concentration in the aqueous phase was determined by an automatic argentometric titration using a Mettler-Toledo DMi141-SC combined silver ring electrode in combination with a Mettler-Toledo T5 Excellence titrator and an InMotion Flex autosampler. Samples were prepared by weighing a calculated amount of sample and diluting it in ca. 40 mL of milliQ water. Hereafter, ca. 2 mL of a 5 vol% triton-X-100 solution was added, the pH was adjusted to approximately 4.5–5.0 with H_2_SO_4_, and the solution was titrated with a calibrated 0.05 M AgNO_3_ titrant.

The hydroxide concentration in the aqueous phase was determined by an automatic potentiometric titration using a Mettler-Toledo DMi111-SC-combined glass pH electrode in combination with a Mettler-Toledo titrator T5 Excellence and an InMotion Flex autosampler. Samples were prepared by weighing a calculated amount of sample and diluting it in ca. 40 mL of milliQ water, and the solution was titrated with a calibrated 0.1 M HNO_3_ titrant.

The co-extraction of sodium ions was monitored by measuring the sodium concentration in the final LiOH product solution by inductively coupled plasma atomic emission spectroscopy (ICP-OES), with a PerkinElmer Avio 500 spectrometer equipped with an axial/radial dual-plasma view, a GemCone High Solids Nebulizer, a baffled cyclonic spray chamber, a 2.0 mm inner diameter alumina injector, and a PerkinElmer Hybrid XLT torch. The emission lines at 588.995 and 610.362 nm were used for the measurement of sodium and lithium, respectively. Yttrium (5 mg L^‒1^) was used as an internal standard and measured at 324.227 nm.

### Lab-Scale Solvent Extraction Tests

For the first solvent extraction step (SX1), equal masses of the organic phase (0.004 mol [A336][Cl] and the alcohol/phenol (molar ratio = 1:1) in a diluent) and the aqueous phase containing (10 M NaOH) were contacted in 20 mL vials at 20 °C for 30 min. Stirring was performed using a MIX 15 eco multi-position stirring plate (2mag AG) at 900 rpm for 30 min. The resulting phases were transferred to a 15 mL centrifuge tube, centrifuged at 4500 rpm for 5 min (Labofuge 200), and separated using long glass Pasteur pipettes. The aqueous phase was titrated with 0.05 M AgNO_3_ to determine the chloride concentration.

For the second solvent extraction step (SX2), equal masses of the organic phase (prepared in SX1) and the aqueous phase containing 0.25 M LiCl were contacted in 20 mL vials at 20 °C for 30 min. After extraction, the two phases were separated, and the aqueous phase was titrated with 0.1 M HNO_3_ to determine the hydroxide concentration. LiOH is known to form Li_2_CO_3_ when it absorbs CO_2_ from the air. Some of the LiOH formed in SX2 was transformed into Li_2_CO_3_ by contact with air dissolved in the aqueous phase. This was evident from the presence of 2 equivalence points in the titration curve. The content of hydroxides and carbonates are determined in an aqueous acid–base titration using 0.1 M HNO_3_ as titrant.

### Mixer–Settler Experiments

Mixer–settler experiments were conducted in PTFE lab-scale mixer–settler units (Rousselet Robatel Model UX 1-1). In each mixer–settler, the effective volumes of the mixer and the settler corresponded to 35 mL and 143 mL, respectively. The settling area corresponded to 49 cm^2^ and PTFE coalescence plates were added to accelerate phase disengagement. Peristaltic pumps (Masterflex L/S®) were used to pump the organic and aqueous phases. For SX1 (preparation of Aliquat 336 phenolate ([A336][OR]), the flow rates were adjusted at 12 mL/min and 6 mL/min for the aqueous and organic phases, respectively. Samples were taken from each stage every 30 min. The chloride concentration was monitored by the titration method with 0.05 M AgNO_3_.

The organic phase containing 0.58 M [A336][OR] in Shellsol D70 obtained from SX1 was fed into SX2 for the conversion of LiCl to LiOH with mixer–settlers in counter-current mode at an organic-to-aqueous volume ratio O/A = 3/1. The flow rate of the aqueous and organic phases corresponded to 2 mL/min and 6 mL/min, respectively. Aqueous samples were taken every 60 min from the aqueous weir of each stage and were titrated with 0.1 M HNO_3_ to determine the LiOH concentration.

### Crystallization of Lithium Hydroxide Monohydrate

The crystallization of LiOH·H_2_O from the final LiOH product solution of the mixer–settler experiment (comprising 1.55 M LiOH and 0.025 M LiCl) was compared using different methods: (1) crystallization via evaporation and (2) antisolvent precipitation. In the first method, water was evaporated at 90 °C until the solid LiOH·H_2_O crystallized out. In the second method, LiOH·H_2_O was recovered from the aqueous solution by adding a known amount of ethanol or isopropanol. The volume ratio of alcohol/aqueous solution was varied in the range 0.5 to 7.0. In both cases, the crystals of LiOH·H_2_O were filtered using a Buchner funnel under reduced pressure on Whatman filter paper (0.45 *µ*), washed with the corresponding solvents, and dried at 110 °C for 3 h.

## Results and Discussion

### Principles of the Conversion Process

The conversion of an aqueous solution of lithium chloride to an aqueous solution of lithium hydroxide is achieved by solvent extraction. The direct chloride–hydroxide anion exchange by a liquid anion exchanger (*i.e.*, a basic extractant) is not an efficient process because of the difficulty to generate a solvent with a high hydroxide concentration. Therefore, two coupled solvent extraction steps are used for the conversion. In the first solvent extraction step (SX1), a solvent comprising an onium chloride (typically a quaternary ammonium chloride), an alcohol or phenol derivative and a diluent is contacted with an aqueous NaOH solution. The alcohol or phenol reacts with the hydroxide ions in the organic phase and is transformed into the corresponding alcoholate or phenolate anion. Simultaneously, the chloride ion of the onium chloride is transferred to the aqueous phase so that electric neutrality of the phases is maintained, resulting in the formation of an onium alcoholate or phenolate dissolved in the organic phase. This organic phase is used as the solvent in the second solvent extraction step (SX2), where it is contacted with an aqueous LiCl solution. The alcoholate or phenolate ion is protonated by reaction with water, thus, yielding the alcohol or phenol in the organic phase. Simultaneously, a chloride ion is transferred from the aqueous phase to the organic phase. This reaction between the alcoholate or phenolate ion and water could either take place at the interphase between the organic and the aqueous phase, or the reaction can occur with extracted water. In the latter case, the hydroxide is formed in the organic phase but is exchanged with a chloride ion in the aqueous phase since the hydroxide ion has a high tendency to be hydrated and to be transferred to the aqueous phase. Thus, after completion of SX2, the starting mixture of the onium chloride and the alcohol or phenol is regenerated. The organic phase is transferred once more to SX1, where it is recontacted with the concentrated sodium hydroxide solution, and the cycle can be repeated.

SX1 can be represented by the following chemical reaction:

1 $$ \left[ {{\text{Q}}^{ + } } \right]\left[ {{\text{Cl}}^{ - } } \right]_{{{\text{org}}}} + {\text{ ROH}}_{{{\text{org}}}} + {\text{ NaOH}}_{{{\text{aq}}}}   \leftrightarrows  \left[ {{\text{Q}}^{ + } } \right]\left[ {{\text{OR}}^{ - } } \right]_{{{\text{org}}}} + {\text{ NaCl}}_{{{\text{aq}}}} + {\text{ H}}_{{2}} {\text{O}}. $$

SX2 comprises the following chemical reaction:

2 $$ \left[ {{\text{Q}}^{ + } } \right]\left[ {{\text{OR}}^{ - } } \right]_{{{\text{org}}}} + {\text{ LiCl}}_{{{\text{aq}}}} + {\text{ H}}_{{2}} {\text{O}}\leftrightarrows\left[ {{\text{Q}}^{ + } } \right]\left[ {{\text{Cl}}^{ - } } \right]_{{{\text{org}}}} + {\text{ ROH}}_{{{\text{org}}}} + {\text{ LiOH}}_{{{\text{aq}}}}. $$

where [Q^+^][Cl^−^] is an onium chloride (quaternary ammonium or phosphonium chloride), ROH is an alcohol or phenol, and [Q^+^][OR^−^] is an onium alcoholate or phenolate, whereas subscript “org” represents species in the organic phase and subscript “aq” represents species in the aqueous phase. Although there is only a single organic phase in the system (same organic phase for SX1 and SX2), the aqueous phases in the reactions represented by Eqs. (1) and (2) comprise different aqueous phases.

The chemical reactions behind the process illustrate the tendency to transfer the least hydrated species to the organic phase in solvent extraction by basic extractants, or alternatively, the transfer of the most easily hydrated species to the aqueous phase [34]. The tendency of the anions to be hydrated follows the following order: alcoholate/phenolate < chloride < hydroxide. The position of the chloride anion in this series is crucial for the process. In SX1, deprotonation of the alcohol/phenol leads to transfer of chloride ions, not of alcoholate/phenolate ions, to the aqueous phase for maintaining the charge balance because chloride ions have a stronger tendency for hydration than alcoholate/phenolate ions. On the other hand, in SX2, the chloride ions are transferred to the organic phase because chloride ions have a weaker tendency to get hydrated than hydroxide ions.

**Fig. 1:**
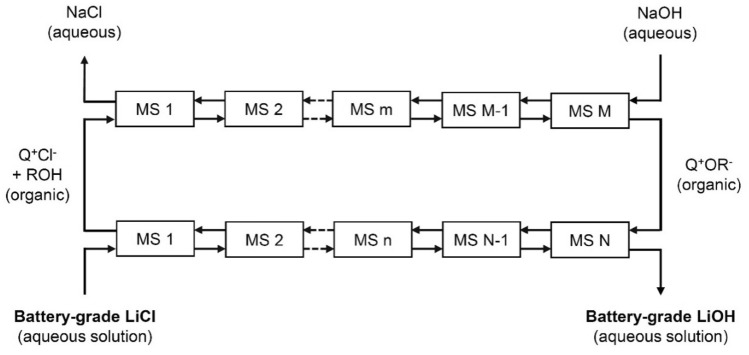
Schematic representation of the conversion of an aqueous LiCl solution into a LiOH solution with two solvent extraction steps, i.e*.*, SX1 at the top and SX2 at the bottom. MS = mixer–settler. Here [Q^+^][Cl^−^] is an onium chloride (quaternary ammonium or phosphonium chloride), ROH is an alcohol or phenol, while [Q^+^][OR^−^] is an onium alcoholate or phenolate

In Fig. 1 it is shown how SX1 and SX2 can be performed in a continuous counter-current mode with mixer–settlers (although other types of contactors could be used as well). It is evident that NaOH is the only chemical that is consumed and that an aqueous NaCl waste stream is generated. In the next sections, it is discussed how the different variables of the process, including the composition of the organic phase, have been optimized.

### Influence of the Composition of the Organic Phase

The most critical component of the organic phase is the ROH compound, either an aliphatic alcohol or an aromatic alcohol (i.e., a phenol). A proper choice of ROH is essential for the overall success of the process. First of all, the water solubility of the compounds should be as low as possible to avoid losses from the organic phase. Therefore, the compounds should be sufficiently lipophilic, i.e., they should have a high value of the logarithm of octanol/water partition coefficient, log *P*. Also the acidity (p*K*_a_) of ROH is of importance, but the preferred acidity values are opposite for SX1 and SX2. For SX1, a higher acidity (lower p*K*_a_) of ROH is preferred, because a more acidic ROH compound is easier to deprotonate. On the other hand, for SX2, a lower acidity (higher p*K*_a_) is advantageous, because in that case the RO^−^ anion is more basic and easier to protonate by reaction with water. Therefore, there are opposite requirements for p*K*_a_ of ROH for the two solvent extraction steps SX1 and SX2, so that it can be predicted that there exists an optimum p*K*_a_ value. Besides log *P* and p*K*_a_, also other factors might play a role, such as the steric hindrance that the OH group is experiencing. A series of primary alcohols, secondary alcohols, diols, and phenol derivatives were considered as ROH component (Table 1).

**Table 1:** Conversion percentage of the first-solvent extraction step (SX1)

Alcohols/phenols	%*E*-SX1 Diluent: GS190	%*E*-SX1 Diluent: D70
1-pentanol	18.7 ± 0.1	19.0 ± 0.1
2-pentanol	14.0 ± 0.1	14.0 ± 0.1
3-pentanol	13.2 ± 0.1	13.2 ± 0.1
1-penten-3-ol	19.4 ± 0.2	19.7 ± 0.1
3-methyl-3-pentanol	12.9 ± 0.3	12.3 ± 0.2
2,4-dimethyl-3-pentanol	13.5 ± 0.1	12.9 ± 0.2
1-hexanol	20.3 ± 0.1	20.1 ± 0.1
2-ethyl-1-hexanol	16.4 ± 0.1	16.6 ± 0.1
1-octanol	19.8 ± 0.2	20.2 ± 0.1
1-decanol	20.0 ± 0.3	20.1 ± 0.7
2-ethyl-1,3-hexanediol	36.7 ± 0.1	36.7 ± 0.2
2-methyl-2,4-pentanediol	44.4 ± 0.3	45.1 ± 0.7
2,5-dimethyl-2,5-hexanediol	25.8 ± 0.3	25.6 ± 0.2
*p*-cresol	78.7 ± 1.1	88.9 ± 0.7
4-*tert*-butylphenol	Extraction mixture solidified	Extraction mixture solidified
2,6-di-*tert*-butylphenol	82.0 ± 0.9	94.8 ± 0.2
2,4,6-trimethylphenol	88.8 ± 1.2	86.1 ± 0.6
2,6-dimethylphenol	88.7 ± 1.9	87.2 ± 0.2

Organic phase: 0.004 M [A336][Cl] and phenol/alcohol (molar ratio = 1:1) in Shell GTL Solvent GS190 or Shellsol D70; Aqueous phase: 10.0 M NaOH; mass ratio = 1:1; temperature = 20 °C; contact time = 30 min

The requirements for the onium chloride are less strict than those for the ROH component. Nevertheless, the onium chloride should be (1) easily soluble in the diluent to avoid third-phase formation and (2) poorly soluble in the aqueous phase to avoid dissolution losses. The most obvious onium chlorides are quaternary ammonium chlorides and quaternary phosphonium chlorides with a long alkyl chain. The ammonium salts have the advantage of having a better chemical stability against concentrated alkali solutions [35]. In this study, we considered two commercially available onium chlorides: Aliquat 336 and Cyphos IL 101. Aliquat 336 is a mixture of quaternary ammonium chlorides with C_8_ (octyl) and C_10_ (decyl) chains, with trioctylmethylammonium chloride as the main component. The quaternary phosphonium chloride Cyphos IL 101 is mainly composed of trihexyl(tetradecyl)phosphonium chloride.

Three different diluents have been tested: Shellsol A150, Shellsol D70, and Shell GTL Solvent GS190. Shellsol A150 is an aromatic diluent (> 99.5% aromatic content). Shellsol D70 consists predominantly of C11–C14 paraffins and naphthenes, with a very low aromatic content. Shell GTL Solvent GS190 is a Fischer–Tropsch solvent (prepared by gas-to-liquid technology), with a boiling range between 187 and 218 °C. It is an aliphatic diluent with 97 wt% iso- and normal paraffins, 3 wt% naphthenes, and < 100 mg/kg aromatics [36]. However, it was observed that the use of Shellsol A150 led to the formation of a precipitate and a change in volume ratio when contacting Shellsol A150 with a concentrated NaOH solution. Therefore, Shellsol A150 was not considered for further extraction studies. Also the use of nonylphenol and 4-*tert*-octylphenol in combination with the diluents Shell GTL Solvent GS190 and Shellsol D70 resulted in gel formation. Hence, no experimental extraction data are reported for these phenols.

The *conversion percentage* for SX1 was defined as follows:

3 $$ \% E{\text{-SX}}1 = \, \left( {\left[ {{\text{Cl}}^{ - } } \right]_{{{\text{aq}}}} \cdot{V}_{{{\text{aq}}}} } \right)/(\left[ {{\text{Cl}}^{ - } } \right]_{{{\text{ini,org}}}} \cdot{V}_{{{\text{org}}}} )\cdot{1}00, $$

where [Cl^−^]_aq_ and [Cl^−^]_ini, org_ are the chloride concentration in the aqueous phase and in the initial organic phase (M); *V*_aq_ and *V*_org_ are the volume of the aqueous and organic phase (L), respectively. The experiments were performed in triplicate and the data are presented in Table 1 as average values with a standard deviation. The conclusion to be drawn from Table 1 is that phenols give much higher [A336][OR] conversion percentage than the primary alcohols, secondary alcohols, and diols. The order of increasing conversion percentage is alcohols < diols < phenols. This order is agreement with the acidity (p*K*_a_ values) of the ROH compounds, as explained earlier in the text: more acidic compounds lead to easier deprotonation. There is little difference between the conversion percentages when comparing the diluents Shell GTL Solvent GS190 and Shellsol D70.

The organic phase containing [A336][OR] in a diluent was used for the second-solvent extraction step to convert LiCl into LiOH. The *conversion percentage* for SX2 was defined as follows:

4 $$ \% E{\text{-SX}}2 = \, \left( {\left[ {{\text{LiOH}}} \right]_{{{\text{eq}}}} /\left[ {{\text{LiCl}}} \right]_{{{\text{ini}}}} } \right)\cdot{1}00, $$

where [LiOH]_eq_ is the LiOH concentration in the aqueous phase and [LiCl]_ini_ is the initial concentration of LiCl in the aqueous feed solution. The experiments were performed in triplicate and the data are presented in Table 2 as average values with a standard deviation. Here the general trend is opposite to the values for SX2; the order of increasing conversion percentage is alcohols > diols > phenols. Once more this is in agreement with the prediction we made earlier in the text: less acidic compounds (higher p*K*_a_ values) lead to higher conversion percentages.

**Table 2:** Conversion percentage of the second-solvent extraction step (SX2)

Alcohols/phenols	%*E*-SX2 Diluent: GS190	%*E*-SX2 Diluent: D70
1-pentanol	87.7 ± 5.4	86.6 ± 0.7
2-pentanol	81.4 ± 0.1	82.8 ± 1.4
3-pentanol	79.4 ± 0.8	81.6 ± 2.5
1-penten-3-ol	78.8 ± 1.9	79.1 ± 4.4
3-methyl-3-pentanol	48.4 ± 3.3	72.6 ± 3.0
2,4-dimethyl-3-pentanol	35.2 ± 1.4	70.8 ± 8.2
1-hexanol	56.7 ± 0.5	79.2 ± 6.5
2-ethyl-1-hexanol	84.1 ± 0.1	80.7 ± 7.7
1-octanol	76.1 ± 9.2	86.7 ± 6.7
1-decanol	71.2 ± 2.5	78.9 ± 6.6
2-ethyl-1,3-hexanediol	69.7 ± 5.0	58.2 ± 3.1
2-methyl-2,4-pentanediol	55.3 ± 2.8	50.6 ± 3.8
2,5-dimethyl-2,5-hexanediol	3-phase system	41.9 ± 0.7
*p*-cresol	31.1 ± 0.2	32.3 ± 2.1
2,6-di-*tert*-butylphenol	72.9 ± 1.2	78.10 ± 2.2
2,4,6-trimethylphenol	25.3 ± 0.6	27.4 ± 0.9
2,6-dimethylphenol	18.7 ± 0.8	21.2 ± 0.9

Organic phase: [A336][OR] and phenol/alcohol (molar ratio = 1:1) in Shell GTL Solvent GS190 or Shellsol D70; Aqueous phase: 0.25 M LiCl; mass ratio = 1:1; temperature = 20 °C; contact time = 30 min

The conversion percentage of the two solvent extraction steps combined (%*E*-Tot) is shown in Table 3 as average values with a standard deviation. From Table 3, it can be concluded that the order of increasing conversion percentage of the double-solvent extraction operation is alcohols < diols < phenols and that the most promising candidate is 2,6-di-*tert*-butylphenol. For this reason, this compound was selected for further experiments. It must be mentioned that 2,6-di-*tert*-butyl-substituted phenols such as 2,6-di-*tert*-butylphenol are known for their antioxidant properties and are used to prevent free-radical-mediated oxidation reactions in organic fluids [37]. For instance, a similar compound, 2,6-di-*tert*-butyl-4-methylphenol (BHT), is used in solvent extraction systems for cobalt–nickel separation to protect the aliphatic diluent against cobalt-catalyzed oxidation reactions [38]. For Table 3, it is also evident that the total conversion percentage was also higher when diluent Shellsol D70 was used instead of Shell GTL Solvent GS190. For this reason, Shellsol D70 was selected for subsequent experiments. Note that Shellsol D70 is a commonly used diluent in solvent extraction processes for metal ions.

**Table 3:** Conversion percentage of the double-solvent extraction operation (SX1, followed by SX2)

Alcohols/phenols	%*E*-Total Diluent: GS190	%*E*-Total Diluent: D70
1-pentanol	16.8 ± 0.9	16.8 ± 0.2
2-pentanol	11.7 ± 0.1	11.9 ± 0.2
3-pentanol	10.7 ± 0.1	11.1 ± 0.3
1-penten-3-ol	15.7 ± 0.3	16.0 ± 0.8
3-methyl-3-pentanol	6.3 ± 0.4	8.9 ± 0.2
2,4-dimethyl-3-pentanol	4.8 ± 0.2	9.4 ± 0.9
1-hexanol	11.5 ± 0.1	16.4 ± 1.3
2-ethyl-1-hexanol	14.2 ± 0.1	13.7 ± 1.2
1-octanol	15.1 ± 1.9	17.5 ± 1.3
1-decanol	14.1 ± 0.7	15.9 ± 0.8
2-ethyl-1,3-hexanediol	11.1 ± 0.8	9.2 ± 0.5
2-methyl-2,4-pentanediol	10.4 ± 0.4	9.8 ± 0.4
2,5-dimethyl-2,5-hexanediol	3-phase system	3.0 ± 2.6
*p*-cresol	24.4 ± 0.2	32.7 ± 1.75
2,6-di-*tert*-butylphenol	59.7 ± 0.4	78.0 ± 1.4
2,4,6-trimethylphenol	22.4 ± 0.8	24.3 ± 0.9
2,6-dimethylphenol	16.6 ± 0.9	18.5 ± 0.8

The experimental conditions can be found in the footnotes to Tables 1 and 2.

**Fig. 2:**
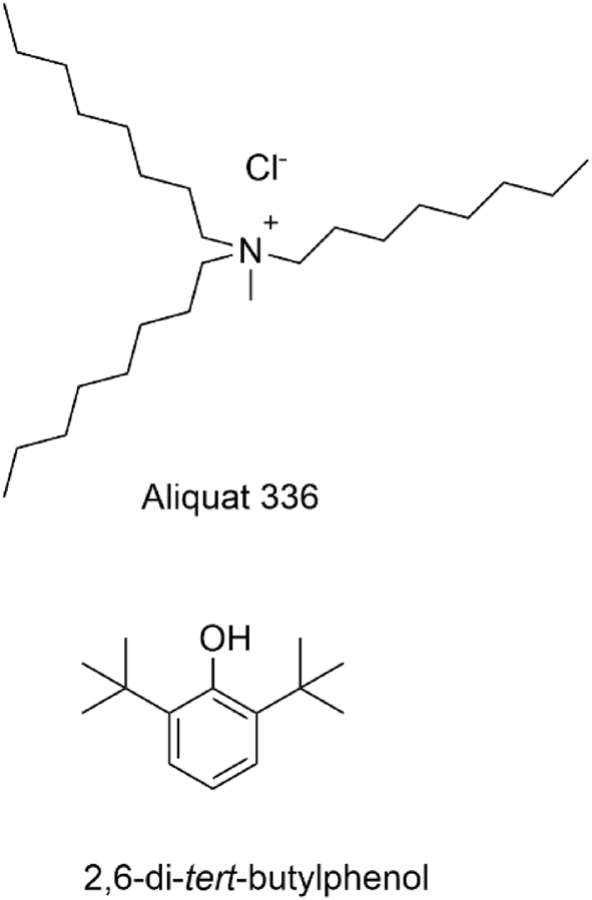
Chemical structure of the quaternary ammonium chloride Aliquat 336 (its main component trioctylmethylammonium choride is shown) and the phenol derivative 2,6-di-*tert*-butylphenol

A double-solvent extraction operation (SX1 + SX2), as described in the previous section, was performed using different onium chlorides and adopting Shellsol D70 as a diluent. The tested onium chlorides were Aliquat 336 and Cyphos IL 101. The experiments were performed in triplicate. The conversion rates for both the first (SX1) and the second (SX2) extraction step, as well as for the combined process, are reported in Table 4 as average values with a standard deviation. Only minor differences were observed between the conversion rates obtained for Aliquat 336 and Cyphos IL 101. For further experiments, Aliquat 336 has been selected as the quaternary ammonium chloride and 2,6-di-*tert*-butylphenol as the phenol derivative (Fig. 2).

**Table 4:** Effect of the onium chloride on the double-solvent extraction operation

Phenol	Onium Cl	%*E*-SX1^a^	%*E*-SX2^b^	%*E*-Total
*p*-cresol	Aliquat 336	88.92 ± 0.7	35.90 ± 1.9	32.7 ± 1.8
	Cyphos IL 101	89.73 ± 0.1	49.27 ± 2.3	41.6 ± 1.9
2,6-di-*tert*-butylphenol	Aliquat 336	94.83 ± 0.2	80.16 ± 1.3	78.0 ± 1.4
	Cyphos IL 101	99.32 ± 0.4	72.40 ± 0.6	67.7 ± 0.8
2,4,6-trimethylphenol	Aliquat 336	86.51 ± 0.6	39.22 ± 5.5	34.8 ± 4.8
	Cyphos IL 101	90.49 ± 0.3	49.29 ± 1.4	42.0 ± 1.2

^a^Organic phase: 0.004 mol Aliquat 336 or Cyphos IL 101 and phenol (molar ratio = 1:1) in Shellsol D70; Aqueous phase: 10.0 M NaOH; mass ratio = 1:1; temperature = 20 °C; contact time = 30 min

^b^Organic phase: Aliquat 336 or Cyphos IL 101 (molar ratio = 1:1) in Shellsol D70; Aqueous phase: 0.25 M LiCl; mass ratio = 1:1; temperature = 20 °C; contact time = 30 min

### Effect of Composition of Aqueous Feed Phases

To study the effect of the type of base in the feed for SX1, a double-solvent extraction operation was performed using 2,6-di-*tert*-butylphenol, *p*-cresol or 2,4,6-trimethylphenol, and Aliquat 336 in the diluent Shellsol D70 as the organic phase, and with either a sodium hydroxide solution (10 M) or an aqueous ammonia solution (15 M) as base. From Table 5, it can be concluded that the conversion rates with the ammonia solution are much lower than when using a sodium hydroxide solution. These low conversion rates might be due to the fact that ammonia is a weak base so that the equilibrium ROH + NH_3(aq)_ ⇆ RO^−^ + NH_4_^+^_(aq)_ is shifted to the left.

**Table 5:** Effect of the base on the SX1

Phenol	%*E*-SX1 10 M NaOH	%*E*-SX1 15 M NH_3_
2,6-di-*tert*-butylphenol	94.8 ± 0.2	14.5 ± 0.1
*p*-cresol	88.9 ± 0.7	49.7 ± 0.1
2,4,6-trimethylphenol	86.5 ± 0.6	40.2 ± 0.3

Organic phase: 0.004 mol [A336][Cl] and phenol (molar ratio = 1:1) in Shellsol D70; Aqueous phase: 10.0 M NaOH or 15 M NH_3_; mass ratio = 1:1; temperature = 20 °C; contact time = 30 min

**Fig. 3:**
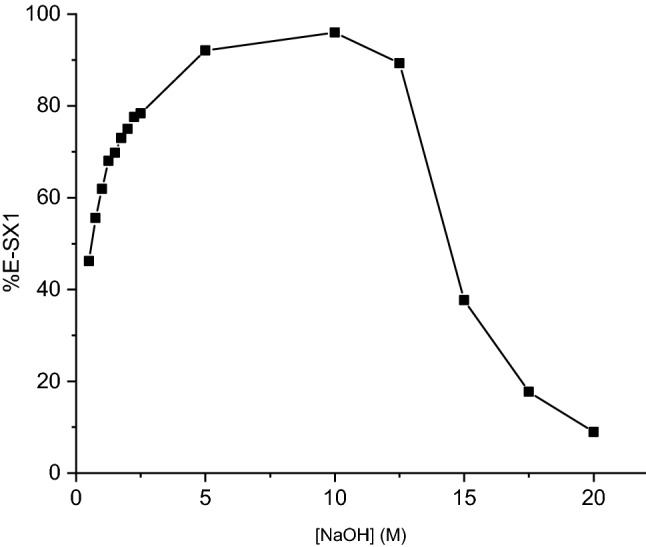
Effect of the NaOH concentration on the extraction efficiency in the first-solvent extraction step (SX1)

The conversion of Aliquat 336, [A336][Cl], to its phenolate form [A336][OR], was also investigated as a function of the NaOH concentration (Fig. 3, Table S1 in the online supplementary material). The conclusion to be drawn is that higher conversions to [A336][OR] were achieved when the NaOH concentration was raised up to 10 M. A further increase in NaOH concentration (> 10 M) led to a decrease in conversion rate, due to the decomposition of the organic phase. Therefore, the optimum concentration of NaOH is preferably less than 2.5 M.

**Fig. 4:**
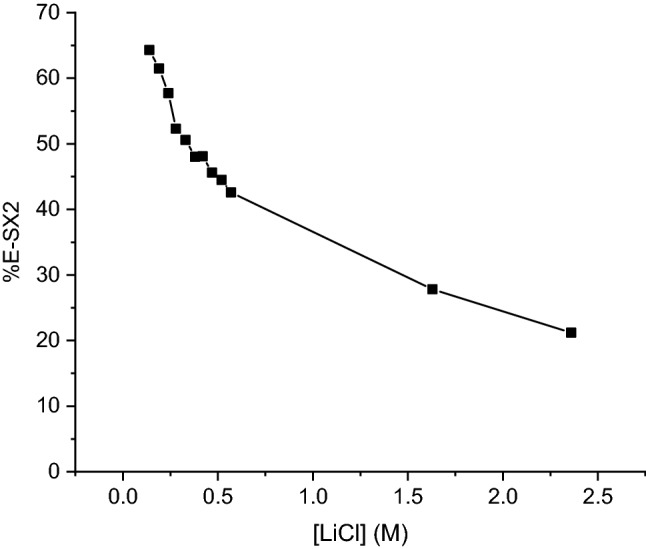
Effect of the LiCl concentration on the extraction efficiency in the second-solvent extraction step (SX2)

The influence of the LiCl concentration on the conversion of LiCl to LiOH was determined for LiCl concentrations from 0.14 to 2.36 M. Equal volumes of the organic phase (0.59 M [A336][OR] and 2,6-di-*tert*-butylphenol (molar ratio = 1:1) in Shellsol D70) and the aqueous phase (0.14–2.36 M LiCl) were contacted. Increasing the LiCl concentration in the aqueous feed reduced the conversion percentage of LiOH (Fig. 4, Table S2).

### Effect of Concentration of Components in the Organic Phase

The effect of the phenol/[A336][Cl] molar ratio was examined in the range from 0.5 to 1.5. The concentration of [A336][Cl] was kept constant at 0.65 M, while the concentration of 2,6-di-*tert*-butylphenol was varied from 0.33 to 1.3 M (Table 6). The conversion yield of SX1 and SX2 enhanced with increasing phenol/[A336][Cl] molar ratio from 0.5 to 1.0. The conversion yield reached a plateau of 88% for SX1 and 52% for SX2 at a phenol/[A336][Cl] molar ratio ≥ 1:1.

**Table 6:** Effect of the phenol/[A336][Cl] molar ratio on the first- and second-solvent extraction step (SX1) and (SX2) and on co-extraction of sodium ions

Phenol/[A336][Cl]	%*E*-SX1	%*E*-SX2	Na^+^ (mg L^−1^)
0.5	51.7	39.8	84.6
0.6	59.1	43.3	69.0
0.7	67.2	47.0	55.5
0.8	72.4	49.5	53.8
0.9	78.0	51.5	54.0
1.0	81.7	52.8	50.7
1.1	85.1	52.5	71.3
1.2	86.7	52.5	115.2
1.3	87.9	52.1	151.2
1.4	88.2	51.0	199.8
1.5	88.9	50.0	210.8

Organic phase: 0.65 M [A336][Cl]; molar ratio 2,6-di-*tert*-butylphenol/[A336][Cl] = 0.5–1.5 in Shellsol D70; Aqueous phase: 2.0 M NaOH (SX1; O/A = 1/2) and 1.65 M LiCl (SX2; O/A = 3/1); temperature = 20 °C; contact time = 30 min

The phenol/[A336][Cl] molar ratio must be monitored as closely as possible to avoid co-extraction of sodium ions. For instance, a molar ratio of phenol/[A336][Cl] of 1:1 yielded the lowest co-extraction of 50 mg L^−1^ Na^+^ as impurity in the final LiOH product solution. On the other hand, if the molar ratios of phenol/[A336][Cl] were higher than 1:1, the more the LiOH solution was contaminated with Na^+^. For instance, 211 mg L^−1^ Na^+^ was co-extracted to the organic phase at a phenol/[A336] molar ratio of 3:2. However, if the phenol/[A336][Cl] molar ratio is too low, only low conversion efficiencies were observed for SX1 and SX2. At the same time, there was a rather high co-extraction of Na^+^ (85 mg/L Na^+^ at a phenol/[A336][Cl] molar ratio of 1:2), due to an increased mutual solubility of aqueous phase in the organic phase. Thus, a molar excess of the phenol derivative with respect to the onium chloride must be avoided, because in that case Na^+^ ions will be co-extracted to the organic phase upon contact of the organic phase with the NaOH solution in SX1. Extraction of sodium by phenols is a phenomenon that has been described in the literature [39].

The conversion of LiOH was investigated as a function of the phenolate concentration [A336][OR] in the organic phase. The conversion percentage of LiOH increased with higher [A336][OR] concentration (Table S3). More than 62% of LiCl was converted to LiOH with 0.59 M [A336][OR] in a single contact. [A336][OR] concentrations in excess of 0.59 M negatively affected the phase separation, decreased the conversion efficiency of LiOH, and increased the co-extraction of Na^+^ as impurity (due to an increased solubility of the aqueous phase in the organic phase).

### Contact Time

The effect of contact time between the organic and aqueous phase in SX1 was studied between 1 and 60 min for the conversion of [A336][Cl] to [A336][OR], using 2.0 M NaOH at O/A = 1/2. SX1 step is fast and efficient. Equilibrium was achieved within 4 min after shaking (Table S4). In fact, a short contact time (< 2 min) between the NaOH solution and the organic phase at 20 °C is preferred to minimize the decomposition of the organic phase.

In the same way, the contact time required for the LiOH conversion was studied in the range 1.0–60 min (SX2). The conversion of LiCl to LiOH is rapid; the equilibrium state was attained within 1 min (Table S5). A short contact time is preferred, particularly during operation of mixer–setters, to minimize the conversion of LiOH to Li_2_CO_3_.

### Batch Counter-Current Extraction

**Fig. 5:**
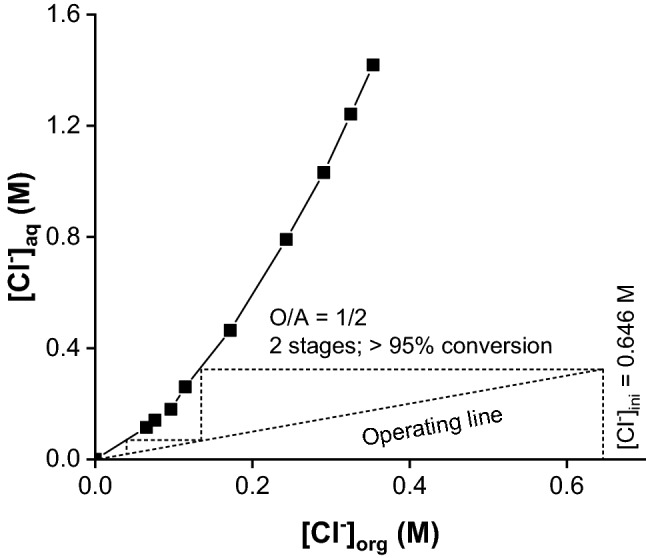
McCabe–Thiele diagram for the first-solvent extraction step (SX1). Organic phase: 0.65 M [A336][Cl] and 2,6-di-*tert*-butylphenol (molar ratio = 1:1) in Shellsol D70; Aqueous phase: 2.0 M NaOH; O/A = 1/5 to 5/1; temperature = 20 °C; contact time = 30 min

The distribution isotherms and McCabe–Thiele diagrams were used to determine the number of theoretical stages for the first and the second steps of the solvent extraction process (SX1 and SX2). For SX1, the influence of O/A volume ratio (organic-to-aqueous volume ratio) on the conversion of [A336][Cl] + ROH in [A336][OR] was investigated for O/A ratios of 1/5 to 5/1, for two NaOH concentrations (1.0 and 2.0 M). A 2.0 M NaOH solution led to a higher conversion to [A336][OR] with respect to a 1.0 M NaOH solution. The McCabe–Thiele diagram shows that at least two counter-current stages are required to convert > 95% of [A336][Cl] + ROH to [A336][OR] at a O/A ratio of 1/2 (Fig. 5, Table S6).

A batch simulation of two-stage counter-current conversion of [A336][Cl] to [A336][OR] was performed to select the optimized conditions that can be applied to the mixer–settlers setup. For the first contact, the fresh organic phase (0.65 M [A336][Cl] and 2,6-di-*tert*-butylphenol at a molar ratio of 1:1 in Shellsol D70 diluent) was mixed with the fresh aqueous solution of 2.0 M NaOH at O/A = 1/2 and 2000 rpm for 4 min. Afterwards, the obtained raffinate was used as the aqueous feed for the second stage where it was contacted with fresh organic phase. Subsequently, the loaded organic phase obtained in the second stage was contacted with fresh aqueous phase. The process was repeated until the steady state was achieved. The final raffinates had a similar chloride concentration and resembled the streams that would exist in an actual continuous counter-current extraction. A high conversion percentage (i.e*.*, [A336][Cl] to [A336][OR]) of > 97% was achieved with a 2-stage counter-current simulation (%*E* = 10.2% after stage 1 and 97.5% after stage 2). This is in agreement with the prediction made using the McCabe–Thiele diagram (Fig. 5).

**Fig. 6:**
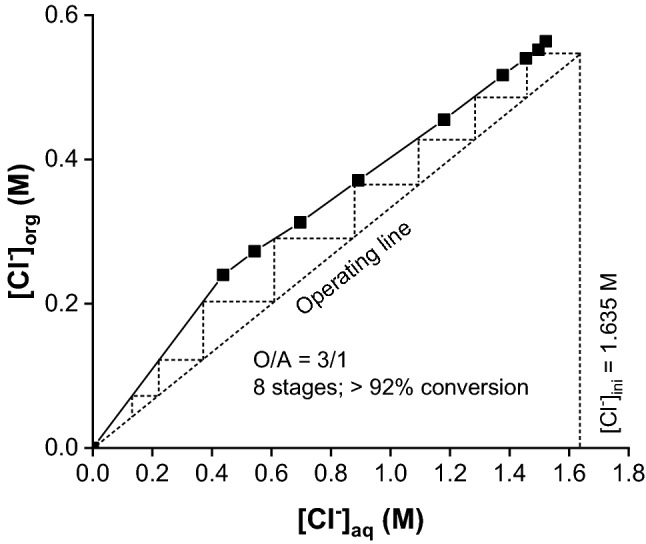
McCabe–Thiele diagram for the second-solvent extraction step (SX2). Organic phase: 0.59 M [A336][OR] in Shellsol D70; Aqueous phase: 1.64 M LiCl; O/A = 1/5 to 5/1; temperature = 20 °C; contact time = 30 min

In a similar way, the dependence of the LiOH conversion on the variation of O/A volume ratio was studied for O/A from 1/5 to 5/1. As expected, a larger organic volume resulted in an increase in LiOH conversion. At a fixed O/A ratio, the conversion percentage of LiOH slightly decreased with increasing LiCl concentration in the feed solution. On the other hand, an increase in [A336][OR] concentration up to 1.0 M led to a slightly higher LiOH conversion but caused problems with the phase separation. For these reasons, an O/A = 3/1 and 0.59 M [A336][OR] were selected for upscaling the SX2 process. The McCabe–Thiele diagram suggested that 8 counter-current stages are required to achieve a LiOH conversion of more than 92% at O/A = 3/1 (Fig. 6, Table S7).

**Fig. 7:**
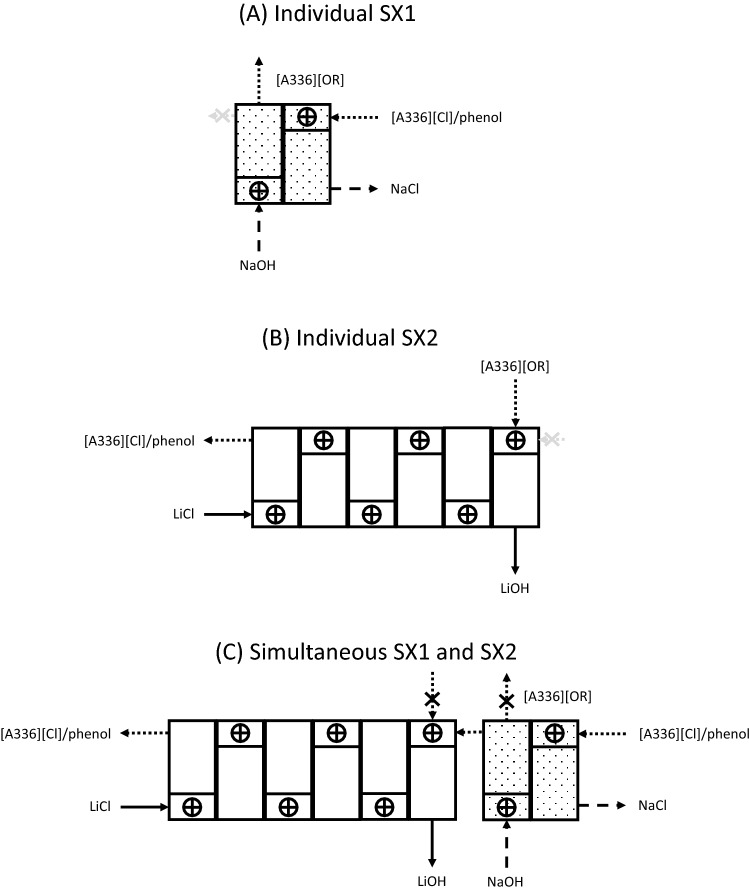
Configuration of the mixer–settlers in the continuous counter-current solvent extraction experiments for **A** individual SX1, **B** individual SX2, and **C** Simultaneous SX1 and SX2

A simulation of a 6-stage batch counter-current extraction for conversion of LiCl into LiOH was performed using 1.64 M and 2.36 M LiCl in the feed solution at O/A = 3/1. The organic phase contained 0.59 M [A336][OR] in Shellsol D70 diluent. Nearly quantitative conversion (> 99.5%) was obtained for the feed solution of 1.64 M LiCl (Table S8). This high conversion yield allows to produce battery-grade LiOH. These results are even superior than those predicted by using the McCabe–Thiele diagram. On the other hand, increasing the LiCl concentration to 2.36 M caused a slightly lower conversion of > 82% LiOH.

### Continuous Counter-Current Solvent Extraction in Mixer–Settlers

Continuous counter-current solvent extraction experiments were conducted in lab-scale mixer–settler units to evaluate the feasibility of upscaling the developed solvent extraction process. The conversion of [A336][Cl] + ROH to [A336][OR] was operated in counter-current mode using two stages in aqueous continuous mode (SX1) (Fig. 7A). The steady state was reached within 2 h. However, the [A336][OR] conversion remained as low as 68% after 8 h of operation. Afterwards, the flow rate was reduced to 6 mL/min for the aqueous phase and 3 mL/min for the organic phase to increase the retention time. A conversion to [A336][OR] in excess of 90% was accomplished after two more hours of operation (Table S9). No precipitation or third-phase formation was observed during the operation of the mixer–settlers. The loaded organic phase at equilibrium was collected and used for the second-solvent extraction conversion of LiCl to LiOH in a second battery of mixer–settlers.

The conversion of LiCl to LiOH was done in continuous counter-current mode in mixer–settlers, using six stages at O/A = 3/1 (SX2) (Fig. 7B). The organic phase containing 0.58 M [A336][OR] in Shellsol D70 diluent was obtained from the mixer–settler experiments for SX1. The conversion percentage increased with time. A steady state was achieved after 5 h of operation with a conversion yield of LiCl to LiOH of more than 81% (Table S10). An attempt to enhance the conversion efficiency of LiOH was made by reducing the flowrate or by increasing the retention time from 4 to 9 min, but the conversion yields were slightly lower.

In a next experiment, the first- and second-solvent extraction steps (SX1) and (SX2) were carried out simultaneously in mixer–settlers. Two batteries of mixer–settlers were connected so that the organic phase obtained in SX1 could immediately enter SX2 (Fig. 7C). The conversion percentage %*E*-SX1 remained stable, whereas %*E*-SX2 increased with time, with a maximum after 6 h of operation. At equilibrium, more than 87.1% of [A336][OR] was formed in SX1 and 98.5% of LiCl was converted to LiOH in SX2 (Table S11). Higher concentrations of LiOH were achieved because the organic phase [A336][OR] was freshly prepared and instantly used in the next step. The conversion of LiOH to Li_2_CO_3_ by reaction of CO_2_ in the air was less than 7%. This result demonstrates that our process is fully capable of producing a highly pure LiOH solution.

Finally, the mixture of [A336][Cl]/phenol obtained after SX2 was used as organic feed solution of SX1, where it was recontacted with a NaOH solution. The %*E*-SX1 for the recycled organic phase was 89.2% (compared to 87.1% for the fresh organic phase), whereas %*E*-SX2 for the recycled organic phase was 98.3% (compared to 98.5% for the fresh organic phase). The conversion of LiOH to Li_2_CO_3_ by reaction of CO_2_ in the air was lower than 8%. This shows it is possible to regenerate and recycle the organic phase during the operation of mixer–settlers, and that the recycled organic phase performed just as well as the fresh one.

Although we have demonstrated that the solvent extraction process can be carried out in mixer–settlers in continuous counter-current mode, it has to be noted that this type of contactor is not ideal for this process. This is mainly because LiOH tends to react with CO_2_ in the atmosphere to Li_2_CO_3_. In addition, crystallization of [A336][OR] was observed once the mixer–settlers were ceased for more than a week. The solid formation caused some trouble when restarting the mixer–settler operation. To minimize this side reaction, closed systems such as columns should be used or, alternatively, the mixer–settlers should be kept under an inert atmosphere.

### Recovery of Lithium Hydroxide Monohydrate from the Aqueous Solution

Solid lithium hydroxide monohydrate, LiOH·H_2_O, can be obtained from the aqueous lithium hydroxide solution that was prepared by the solvent extraction process. The most straightforward approach might seem to be the removal of the water through evaporation. This method was tested on the final solution obtained from the mixer–settler experiment (1.55 M LiOH and 0.025 M LiCl) by evaporation of the water at 90 °C until the solid LiOH·H_2_O crystallized out. The crystals of LiOH·H_2_O were filtered on a Buchner funnel, washed with cold water (5 °C), and dried at 110 °C for 3 h. More than 87% LiOH·H_2_O was crystallized by evaporation of water. The mass loss is largely caused by the washing step. The purity of LiOH·H_2_O was 99.8%.

A second method was tested for the recovery of LiOH·H_2_O from the aqueous solution, i.e., *antisolvent precipitation* (*drowning-out crystallization*), by addition of a water-miscible solvent in which LiOH·H_2_O is not soluble. It is known that LiCl is well soluble in water–ethanol mixtures, whereas LiOH·H_2_O is not, and addition of ethanol to a LiCl solution has been used to crystallize out LiOH·H_2_O [40]. Ethanol can be recovered from the mother liquor by distillation. Isopropanol (isopropyl alcohol), in addition to ethanol, was tested as antisolvent. The advantage of using isopropanol is that the solubility of LiOH·H_2_O in isopropanol (0.11 g/100 mL at 20 °C) is much lower than that in ethanol (2.18 g/100 mL at 20 °C) [41]. Very high recovery yields of LiOH·H_2_O from the aqueous solution were achieved by addition of isopropanol (up to 95%), but not of ethanol (less than 5%) (Table S12). The recovery yield was found to be dependent on the isopropanol-to-water volume ratio (Table S13). A recovery yield of 94.6% and a purity of 99.8% was obtained for a *V*_alcohol_/*V*_aq_ volume ratio of 7.0.

The antisolvent precipitation was not further optimized in this work, as the target was to merely provide the proof of principle. However, it can be assumed that higher recovery yields can be accomplished by partial evaporation of the water, to increase the LiOH concentration in the solution prior to addition of isopropanol. This partial evaporation of water is also required to induce antisolvent precipitation with ethanol instead of isopropanol as antisolvent. Well-established techniques are available for the recovery of isopropanol from aqueous effluents in the chemical and pharmaceutical industries. The most important methods are azeotropic distillation, extractive distillation, and pervaporation. Especially the membrane-based pervaporation technology is promising for application to our process [42].

### Conceptual Flowsheet

**Fig. 8:**
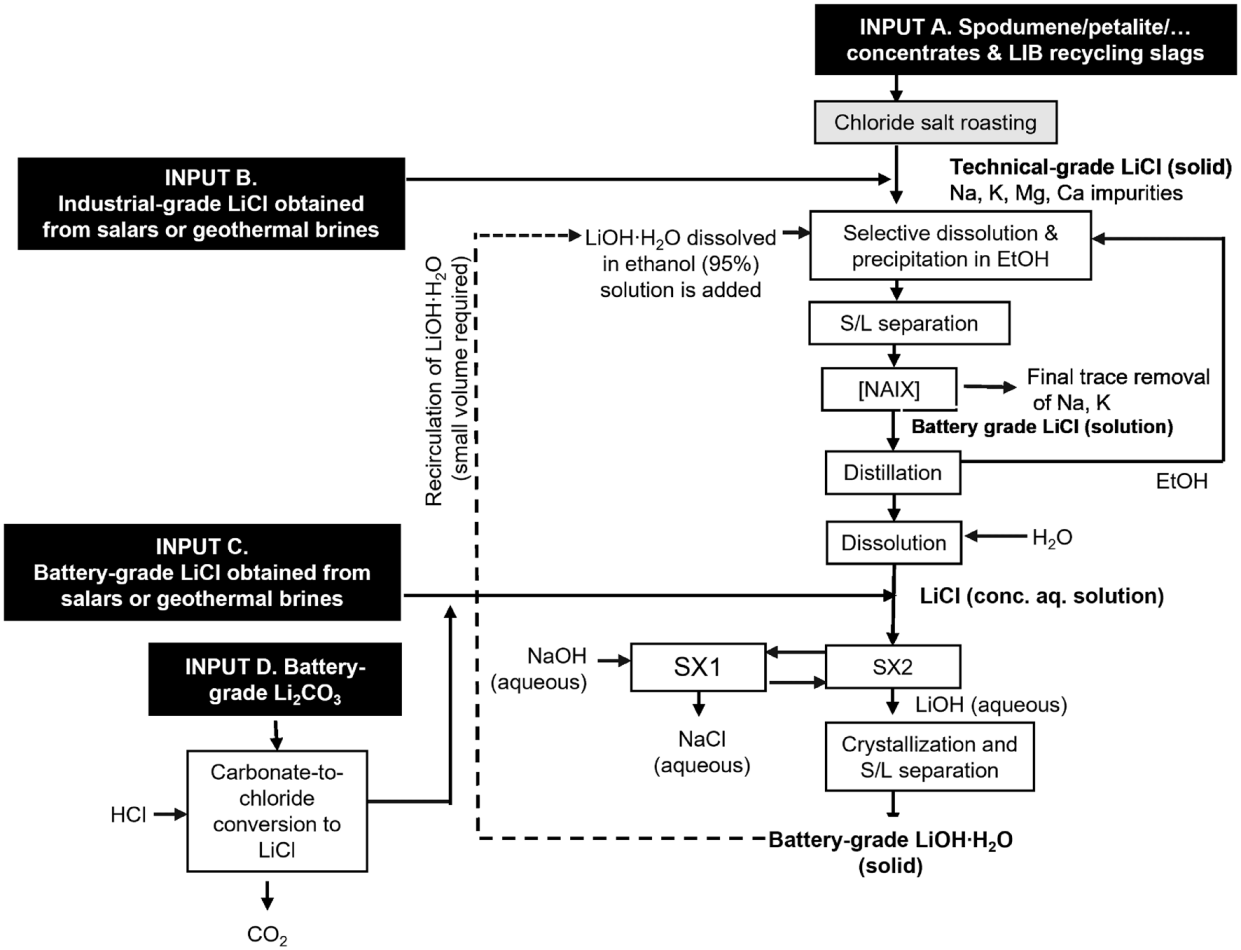
Flowsheet showing the different steps of the process to convert LiCl into LiOH·H_2_O. SX1 and SX2 represent the part of the flowsheet that is described in the present paper. The LiCl used as input for the process can originate from different sources (see text). The option is shown in which LiOH·H_2_O is recovered from the solution by evaporation/crystallization. In the case of antisolvent precipitation, a solvent recovery unit process must be added

The solvent extraction process described in this paper to convert LiCl into LiOH is part of a larger research effort to develop a new flowsheet to purify technical-grade LiCl and transform it into battery-grade LiOH·H_2_O. The flowsheet includes the following steps: (1) dissolving technical-grade LiCl in an organic solvent to obtain a pre-purified LiCl solution, since the solubility of LiCl in organic solvents is significantly higher than the solubility of NaCl or KCl; (2) addition of a solution of an alkali metal hydroxide in an organic solvent; (3) optionally further purifying the LiOH in the organic solution by non-aqueous ion exchange (NAIX); (4) removing the organic solvent from the purified LiCl, followed by dissolution of the LiCl in water; (5) converting LiCl to LiOH by solvent extraction; and (6) crystallizing battery-grade LiOH·H_2_O from the aqueous LiOH solution (Fig. 8). Steps (1) to (4) have been developed in the form of a solvometallurgical process that has been described in a recent paper by our research group [24], whereas steps (5) and (6) are the topic of present paper.

Different input streams can be used for the LiCl required as feed in this flowsheet. These include (1) LiCl from hard-rock lithium ores (pegmatites comprising spodumene, petalite, lepidolite, amblygonite, triphylite, and other lithium minerals) and Li-containing metallurgical slags from pyrometallurgical recycling processes of end-of-life lithium-ion batteries (Input A in Fig. 8), (2a) impure LiCl from salt lake brines and geothermal brines (Input B), as well as (2b) purified LiCl from salt lake brines and geothermal brines (input C). The difference between Input B and Input C is that impure LiCl must be purified first via the solvometallurgical process disclosed in reference [24], whereas input C can be used directly as a feed for the LiCl to LiOH conversion process described in the present paper. For the LiCl to LiOH conversion process, also battery-grade Li_2_CO_3_ can be used as feed, but this must be first dissolved in hydrochloric acid to prepare a LiCl solution (Input D). This is an alternative method to convert Li_2_CO_3_ into LiOH·H_2_O and complements the conversion with Ca(OH)_2_ [6, 7].

Of all the different input streams mentioned in the previous paragraph, Input A is by far the most preferred one, because it is very suitable as a feed for the solvometallurgical process for purification of LiCl [24]. The reason is that purification of LiCl by organic solvents requires solid technical-grade LiCl as input stream. This is the case for Input A if the metallurgical processes for treatment of lithium-containing hard-rock ores or slags from pyrometallurgical recycling processes for Li-ion batteries are operated in such a way that impure LiCl is volatilized. For instance, if spodumene is mixed with CaCl_2_ and the mixture is heated at 800–1200 °C at a pressure of 20 mm Hg or less [43]. The volatile LiCl is condensed from the vapor and collected. This LiCl is already very low in impurities and is very suitable for treatment via the solvometallurgical process. LiCl can be fumed out of molten lithium-containing slags from lithium-ion battery recycling processes [18]. On the other hand, LiCl from solars or geothermal brines are less suitable for this process, because these aqueous streams first need to be evaporated to dryness and, moreover, the solid obtained in that way will not only contain LiCl but also very large concentrations of alkali and alkaline earth impurities. If no solar energy can be used, the evaporation of brines is energy intensive. Moreover, the solvometallurgical process becomes less efficient if the LiCl is very impure.

So far, we have presented only the proof-of-principle of this new flowsheet for purification of LiCl and for conversion of LiCl into LiOH·H_2_O. It is obvious that upscaling experiments are required to bring this process to a higher technological readiness level (TRL) [44], and that these experiments need to be complemented with flowsheet modeling with materials and energy balances, as well as by life-cycle assessment (LCA) and a techno-economical assessment (TEA). However, these studies are outside the scope of the present paper.

Sasson and co-workers have described a liquid membrane system that shows some similarities with our solvent extraction system since their system is also based on a quaternary ammonium chloride and a weakly acidic ROH compound [32, 33]. However, there are some major differences. First of all, these authors state that the efficiency of the chloride/hydroxide exchange system shows the following order for the ROH compounds: diols > primary alcohols > secondary alcohols. We demonstrate that phenol derivatives perform much better than any alcohol or diol. The hexane used by Sasson and co-workers is not suitable as diluent for solvent extraction because of its low flashpoint (−22 °C). We work at much lower NaOH hydroxide concentrations so that there is less danger for chemical decomposition of the quaternary ammonium compound. By using continuous counter-current extraction, we make use of the chemical reagents in the most efficient way, while obtaining enhanced conversion rates.

## Conclusions

This paper reveals how a solution of lithium chloride can be efficiently converted into a solution of lithium hydroxide by a two-step solvent extraction process. Rather than a direct exchange of hydroxide ions by chloride ions through anion exchange, an indirect route is followed to overcome the difficulty to create an organic phase with a high concentration of hydroxide ions. By using a mixture of a quaternary ammonium chloride and a phenol derivative in an aliphatic diluent, an efficient solvent extraction process can be designed. In a first step, the phenol derivative in the organic phase is deprotonated by contact with a sodium chloride solution and a quaternary ammonium phenolate is formed, with simultaneous transfer of chloride ions from the organic to the aqueous phase. In the second step, this organic phase is contacted with an aqueous lithium chloride solution. The water molecules in this aqueous solution act as Brønsted acid towards the phenolate anion that is a strong Brønsted base, and the phenolate is converted to the corresponding phenol. The hydroxide ion in the organic phase is transferred to the aqueous phase, while at the same time, a chloride ion is transferred to the organic phase. This closes the cycle since the original mixture of ammonium chloride and phenol is reformed in the organic phase.

The double SX process was optimized and demonstrated at lab scale using mixer–settlers. It was found that the lithium hydroxide could be recovered from the aqueous solution by evaporation of the water, or, even better, by antisolvent precipitation through addition of isopropanol as antisolvent. Lithium hydroxide monohydrate could be obtained in high purity and high yield. No waste is produced, with the exception of an aqueous NaCl solution. Furthermore, it was shown for two complete cycles that the quaternary ammonium chloride and the phenol can be regenerated to close the materials loop. This method has a general applicability, as long as a highly pure aqueous lithium chloride solution is available. Lithium chloride can originate from different sources, including hard-rock lithium ores, solars, geothermal brines as well as slags obtained from pyrometallurgical recycling of lithium-ion batteries. The process for LiCl to LiOH conversion is compatible with a new solvometallurgical process for purification of technical-grade LiCl to battery-grade LiCl [24]. Further research and development on this new two-step solvent extraction process will go into two directions; first of all, a fundamental study towards a better understanding of the solvent extraction mechanism and, second, upscaling studies to a higher technology readiness level (TRL).

## Supplementary Information

Below is the link to the electronic supplementary material.

Supplementary file1 (PDF 223 kb)
