# Supplementary material for: Conversion of Lithium Chloride into Lithium Hydroxide by Solvent Extraction
Source: J Sustain Metall. 2022 Dec 5;9(1):107–22. doi: 10.1007/s40831-022-00629-2 (PMC10015601; doi:10.1007/s40831-022-00629-2)
Supplement: Supplementary file 1 — Supplementary file1 (PDF 223 kb) [file 40831_2022_629_MOESM1_ESM.pdf]

Supplementary Material for: *Journal of Sustainable Metallurgy*

Conversion of Lithium Chloride into Lithium Hydroxide by Solvent Extraction

Viet Tu Nguyen[1] • Clio Deferm[1] • Ward Caytan[1] • Sofía Riaño[1] • Peter Tom Jones[2] • Koen Binnemans[1*]

✉ Koen Binnemans

Koen.Binnemans@kuleuven.be

[1] KU Leuven, Department of Chemistry, Celestijnenlaan 200F - box 2404, B-3001 Heverlee (Belgium)

[2] KU Leuven, Department of Materials Engineering, Kasteelpark Arenberg 44, P.O. box 2450, B-3001 Heverlee (Belgium)

Table S1. Effect of the NaOH concentration on the first solvent extraction step (SX1)§

NaOH (M)	%*E*-SX1
0.50	46.2
0.75	55.6
1.00	61.9
1.25	68.0
1.50	69.8
1.75	73.0
2.00	75.0
2.25	77.6
2.50	78.4
5.00	92.1
10.0	96.0
12.5	89.3
15.0	37.7
17.5	17.7
20.0	8.94

§Organic phase: 0.65 M (or 30% wt.)[A336][Cl] and 2,6-di-*tert*-butylphenol (molar ratio = 1:1) in Shellsol D70; Aqueous phase: 0.5–20 M NaOH; O/A = 1; temperature = 20 °C; contact time = 30 min.

Table S2. Effect of the LiCl concentration on the second solvent extraction step (SX2)§

LiCl (M)	%*E*-SX2
0.14	64.3
0.19	61.5
0.24	57.7
0.28	52.3
0.33	50.6
0.38	48.0
0.42	48.1
0.47	45.6
0.52	44.5
0.57	42.6
1.63	27.8
2.36	21.2

§Organic phase: 0.59 M [A336][OR] in Shellsol D70; Aqueous phase: 0.14–2.36 M LiCl; O/A = 1/1; temperature = 20 °C; contact time = 30 min.

Table S3. Effect of the [A336][OR] concentration on the second solvent extraction step (SX2)§

[A336][OR]	%*E*-SX2
0.29	20.3
0.32	26.9
0.36	30.7
0.40	37.8
0.47	48.8
0.53	51.5
0.55	53.7
0.57	58.3
0.59	62.9

§Organic phase: 0.29–0.59 M [A336][OR] in Shellsol D70; Aqueous phase: 0.2 M LiCl; O/A = 1/1; temperature = 20 °C; contact time = 30 min.

Table S4. Effect of the contact time on the first solvent extraction step (SX1)[§]

Time (min)	%*E*-SX1
1	61.3
2	72.4
4	85.1
6	86.7
8	87.0
10	86.7
12	86.7
14	87.0
16	87.0
18	87.0
20	87.3
30	87.6
40	87.9
50	87.9
60	88.5

[§]Organic phase: 0.65 M [A336][Cl] and 2,6-di-*tert*-butylphenol (molar ratio = 1:1) in Shellsol D70; Aqueous phase: 2.0 M NaOH; O/A = 1/2; temperature = 20 °C.

Table S5. Effect of the contact time for the second solvent extraction step (SX2)[§]

Time (min)	%*E*-SX2
1.0	54.4
2.0	55.4
4.0	55.7
6.0	56.8
8.0	57.3
10	57.3
20	57.4
30	57.4
60	57.5

[§]Organic phase: 0.59 M [A336][OR] in Shellsol D70; Aqueous phase: 1.64 M LiCl; O/A = 3/1; temperature = 20 °C.

Table S6. Effect of the O/A volume ratio on the first solvent extraction step (SX1)§

O/A volume ratio	%*E*-SX1	
	1.0 M NaOH	2.0 M NaOH
1/5	79.8	89.9
1/4	78.5	88.2
1/3	75.6	85.1
1/2	70.8	82.3
1/1	59.2	73.4
2/1	44.7	62.4
3/1	37.3	55.0
4/1	31.6	49.7
5/1	28.2	45.3

§Organic phase: 0.65 M [A336][Cl] and 2,6-di-*tert*-butylphenol (molar ratio = 1:1) in Shellsol D70; Aqueous phase: 1.0 or 2.0 M NaOH; O/A = 1/5 to 5/1; temperature = 20 °C; contact time = 30 min.

Table S7. Effect of the O/A volume ratio on the second solvent extraction step (SX2)§

O/A volume ratio	%*E*-SX2		
	1.64 M LiCl	2.36 M LiCl	2.36 M LiCl
1/5	6.91[a]	4.88[a]	7.04[b]
1/4	8.44[a]	5.81[a]	8.78[b]
1/3	11.0[a]	7.70[a]	11.0[b]
1/2	15.8[a]	11.3[a]	15.2[b]
1/1	27.8[a]	21.2[a]	26.2[b]
2/1	45.4[a]	36.3[a]	40.9[b]
3/1	57.4[a]	47.0[a]	50.4[b]
4/1	66.7[a]	55.6[a]	57.3[b]
5/1	73.2[a]	62.3[a]	64.0[b]

§Organic phase: [a]0.59 M and [b]1.00 M [A336][OR] in Shellsol D70; Aqueous phase: 1.64 and 2.36 M LiCl; O/A = 1/5 to 5/1; temperature = 20 °C; contact time = 30 min.

Table S8. Simulation of 6-stage counter-current for the second solvent extraction step (SX2)§

Stage	%*E*-SX2	
	1.64 M LiCl	2.36 M LiCl
1	13.4	3.23
2	28.0	4.37
3	44.2	19.3
4	61.8	34.5
5	81.4	55.8
6	99.5	82.0

§Organic phase: 0.59 M [A336][OR] in Shellsol D70; Aqueous phase: 1.64 and 2.36 M LiCl; O/A = 3/1; temperature = 20 °C; contact time = 4 min.

Table S9. Effect of the equilibration time in the individual operation of the first solvent extraction step (SX1) in 2-stage counter-current mixer-settlers§

	%*E*-SX1	
Time (h)	Stage 1	Stage 2
0.3	28.5	38.4
0.5	28.8	58.3
0.7	22.6	63.6
0.8	23.6	63.3
1.0	26.4	66.7
2.0	25.4	68.2
3.0	26.0	68.2
4.0	27.9	66.4
8.0	29.5	65.7
9.0	27.3	65.1
10.0	26.7	77.5
10.5	29.1	90.8
11.0	20.5	91.5
12.0	20.5	91.2
12.5	22.9	90.8
13.0	22.9	90.5

§Organic phase: 0.65 M [A336][Cl] and 2,6-di-*tert*-butylphenol (molar ratio = 1:1) in Shellsol D70; Aqueous phase: 2.0 M NaOH; O/A = 1/2; temperature = 20 °C; retention time = 2–4 min; 1000 rpm.

Table S10. Effect of the equilibration time in the individual operation of the second solvent extraction step (SX2) in 6-stage counter-current mixer-settlers§

	%*E*-SX2					
Time (h)	Stage 1	Stage 2	Stage 3	Stage 4	Stage 5	Stage 6
0.5	8.3	15.3	21.0	24.2	26.4	45.1
1.0	7.2	14.6	20.4	24.5	34.8	56.8
2.0	6.6	13.6	21.8	31.3	48.8	70.8
3.0	7.0	15.0	25.0	37.9	56.5	76.3
4.0	7.6	16.6	27.8	41.0	59.3	81.1
5.0	9.3	19.5	31.4	45.4	63.1	82.8
6.0	9.8	22.0	34.4	48.8	66.2	84.8
7.0	10.9	22.8	35.4	49.7	68.0	85.7
8.0	12.1	24.3	37.7	52.6	69.2	86.3
9.0	13.0	25.1	37.6	52.2	67.3	83.8
10	12.8	26.5	39.0	51.7	67.0	84.4
11	12.8	25.4	40.0	55.3	67.0	83.1
12	11.2	23.7	38.4	53.6	66.7	81.8

§Organic phase: 0.58 M [A336][OR] in Shellsol D70; Aqueous phase: 1.64 M LiCl; O/A = 3/1; 20 °C; retention time = 4–9 min; 1000 rpm

Table S11. Contact time for the simultaneous operation of the first and second solvent extraction step (SX1) and (SX2) in mixer-settlers§

Time (h)	%*E*-SX1	%*E*-SX2
1.0	87.3	26.1
2.0	87.9	59.7
3.0	87.3	75.6
4.0	89.0	84.4
5.0	87.6	89.1
6.0	86.8	93.3
7.0	86.5	95.7
8.0	86.8	95.9
9.0	87.1	98.5

§(SX1) Organic phase: 0.65 M [A336][Cl] and 2,6-di-*tert*-butylphenol (molar ratio = 1:1) in Shellsol D70; Aqueous phase: 2.0 M NaOH; O/A = 1/2; 20 °C; retention time in each mixer = 2 min; 1500 rpm; (SX2) Organic phase: 0.58 M [A336][OR] in Shellsol D70; Aqueous phase: 1.64 M LiCl; O/A = 3/1; temperature = 20 °C; retention time in each mixer = 4 min; 1500 rpm

Table S12. Crystallization of lithium hydroxide monohydrate from the final product solution§

	Evaporation	Antisolvent precipitation	
Method	Water	Ethanol	Isopropanol
Recovery yield (%)	87.3	3.87	94.6
Purity (%)	99.8	100	99.8

§Final product solution: 1.55 M LiOH and 0.025 M LiCl.

Table S13. Effect of volume ratio $V_{alcohol}/V_{aq}$ on the mass recovery of lithium hydroxide (as the monohydrate)

$V_{alcohol}/V_{aq}$	Mass recovery (%)	
	Ethanol	Isopropanol
0.5	6.32	5.94
1.0	6.06	6.06
1.5	5.81	6.39
2.0	5.29	9.30
3.0	4.19	50.6
4.0	4.13	77.5
5.0	4.45	86.7
6.0	3.16	92.4
7.0	3.87	94.6
